# Comparative analysis of plant isochorismate synthases reveals structural mechanisms underlying their distinct biochemical properties

**DOI:** 10.1042/BSR20171457

**Published:** 2018-03-09

**Authors:** Shohei Yokoo, Seiya Inoue, Nana Suzuki, Naho Amakawa, Hidenori Matsui, Hirofumi Nakagami, Akira Takahashi, Ryoichi Arai, Shinpei Katou

**Affiliations:** 1 Faculty of Agriculture, Shinshu University, Minamiminowa 8304, Nagano 399-4598, Japan; 2 Graduate School of Environmental and Life Science, Okayama University, Okayama, Okayama 700-8530, Japan; 3 Plant Proteomics Research Unit, RIKEN CSRS, Yokohama, Kanagawa 230-0045, Japan; 4 Max Planck Institute for Plant Breeding Research, Cologne 50829, Germany; 5 Division of Plant and Microbial Sciences, Institute of Agrobiological Sciences, NARO, Tsukuba, Ibaraki 305-8602, Japan; 6 Research Center for Fungal and Microbial Dynamism, Shinshu University, Minamiminowa 8304, Nagano 399-4598, Japan; 7 Faculty of Textile Science and Technology, Shinshu University, Ueda, Nagano 386-8567, Japan

**Keywords:** isochorismate synthase, salicylic acid, resistance, phytopathology

## Abstract

Isochorismate synthase (ICS) converts chorismate into isochorismate, a precursor of primary and secondary metabolites including salicylic acid (SA). SA plays important roles in responses to stress conditions in plants. Many studies have suggested that the function of plant ICSs is regulated at the transcriptional level. In *Arabidopsis thaliana*, the expression of *AtICS1* is induced by stress conditions in parallel with SA synthesis, and *AtICS1* is required for SA synthesis. In contrast, the expression of *NtICS* is not induced when SA synthesis is activated in tobacco, and it is unlikely to be involved in SA synthesis. Studies on the biochemical properties of plant ICSs are limited, compared with those on transcriptional regulation. We analyzed the biochemical properties of four plant ICSs: AtICS1, NtICS, NbICS from *Nicotiana benthamiana*, and OsICS from rice. Multiple sequence alignment analysis revealed that their primary structures were well conserved, and predicted key residues for ICS activity were almost completely conserved. However, AtICS1 showed much higher activity than the other ICSs when expressed in *Escherichia coli* and *N. benthamiana* leaves. Moreover, the levels of AtICS1 protein expression in *N. benthamiana* leaves were higher than the other ICSs. Construction and analysis of chimeras between AtICS1 and OsICS revealed that the putative chloroplast transit peptides (TPs) significantly affected the levels of protein accumulation in *N. benthamiana* leaves. Chimeric and point-mutation analyses revealed that Thr^531^, Ser^537^, and Ile^550^ of AtICS1 are essential for its high activity. These distinct biochemical properties of plant ICSs may suggest different roles in their respective plant species.

## Introduction

**Figure 1:**
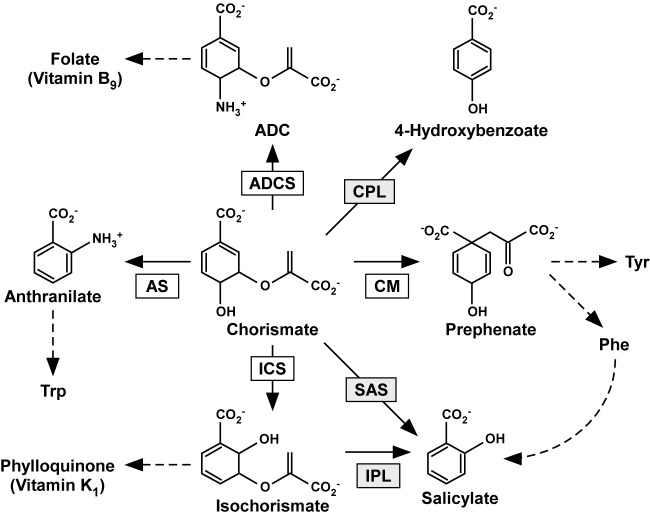
Metabolism of chorismate in plants and microorganisms Chorismate is a branch-point compound for the production of primary and secondary compounds such as aromatic amino acids and vitamins. Enzymes present in both plants and microorganisms, and those found only in bacteria are shown in white and gray boxes, respectively. Abbreviations: ADC, 4-amino-4-deoxychorismate; ADCS, ADC synthase; AS, anthranilate synthase; CM, chorismate mutase; CPL, chorismate pyruvate lyase.

Chorismate, a product of the shikimate pathway, is a common intermediate for the biosynthesis of primary and secondary metabolites in plants and microorganisms [1]. Various types of enzymes use chorismate as a substrate and convert it into other compounds, and the reactions are often important steps toward the production of specific metabolites (Figure 1). Amongst chorismate-utilizing enzymes, anthranilate synthase, isochorismate synthase (ICS), and salicylate synthase (SAS) evolutionally originate from a common ancestor because their overall tertiary structures are highly conserved [2], and they have been grouped together as the MST (menaquinone, siderophore, and tryptophan) superfamily. ICS reversibly converts chorismate into isochorismate in an Mg^2+^-dependent manner. In bacteria, isochorismate is a precursor of siderophores, low molecular weight Fe^3+^ chelators, and menaquinones, electron acceptors. *Escherichia coli* has two *ICS* genes, *EntC* and *MenF*, which are associated with the production of siderophores and menaquinones, respectively [3]. In siderophore production, isochorismate is converted into salicylic acid (SA) or 2,3-dihydroxybenzoic acid (DHBA), which are incorporated into the siderophores. For example, isochorismate pyruvate lyase (IPL), encoded by *PchB*, converts isochorismate into SA in *Pseudomonas aeruginosa* [4], whereas isochorismatase and 2,3-dihydro-2,3-dihydroxybenzoate dehydrogenase, encoded by *EntB* and *EntA*, respectively, successively convert isochorismate into DHBA in *E. coli* [5]. In addition, bifunctional SAS encoded by *Irp9* successively converts chorismate into SA via isochorismate in *Yersinia enterocolitica* [6].

In plants, isochorismate is a precursor of phylloquinone, also known as vitamin K_1_. Phylloquinone functions as an electron acceptor in the photosystem I complex, and it is also essential for human health [7]. Similar to bacteria, isochorismate is also a precursor of SA and DHBA in some plant species [8,9]; however, incorporation of SA or DHBA into siderophores has not been reported currently. In plants, SA functions as a signaling molecule to induce responses against various forms of environmental stress, and its biosynthesis is activated in response to stress in many plant species [10]. Although the biosynthesis pathways of SA in plants are still unclear, ICS plays an important role in the production of SA in some plant species. In *Arabidopsis thaliana*, two genes (*AtICS1* and *AtICS2*) encode ICS, and *AtICS1* plays a key role in stress-induced SA production. In response to pathogen inoculation or ozone exposure, the levels of *AtICS1* transcripts and SA increase in parallel, and *AtICS1* is required for this SA synthesis [11,12]. Moreover, *AtICS1* transcription is also subjected to positive regulation by SA [13]. On the other hand, the expression of *AtICS2* is not associated with SA synthesis [11,14], and it is not required for SA production induced by UV irradiation [15]. Similar to *AtICS1*, a *Nicotiana benthamiana ICS* gene (*NbICS*) is transcriptionally induced by a pathogen elicitor [16] and is required for SA production induced by biotic and abiotic stress conditions [17]. In contrast with *Arabidopsis* and *N. benthamiana*, ICS in tobacco (*Nicotiana tabacum*; *NtICS*) is unlikely to be involved in stress-induced SA production. The transcript levels of *NtICS* were not increased, and ICS activity was not detected after the activation of SA production by tobacco mosaic virus inoculation or ozone exposure [18,19]. Instead, the phenylpropanoid pathway initiated by phenylalanine ammonia lyase was activated and SA was produced in such stress conditions [18–20]. These studies suggested that the function of plant ICSs is regulated mainly at the transcriptional level. One interesting feature of SA is that its basal concentrations differ depending on the plant species. For example, rice (*Oryza sativa*) constitutively contains two to three orders of magnitude higher levels of SA than *Arabidopsis, N. benthamiana*, and tobacco [21]. Although the involvement of rice ICS (OsICS) in SA synthesis is unclear, if such higher levels of SA production are catalyzed by OsICS, its activity might be much higher than AtICS1, NbICS, and NtICS.

The biochemical properties of bacterial ICSs have been studied extensively. For example, the tertiary structures of EntC and MenF have been determined, and many amino acid residues essential or important for ICS activity have been identified [22,23]. In contrast, reports on the biochemical analysis of plant ICSs are limited to that of *Catharanthus roseus* (CrICS) purified from elicited cell cultures [24], and those of AtICS1 and AtICS2 expressed in *E. coli* showed very similar catalytic characteristics [25,26]. In the present study, we analyzed and compared the biochemical properties of AtICS1, NtICS, NbICS, and OsICS expressed in *E. coli* and *N. benthamiana* leaves. Although their primary structures are similar and most key amino acid residues are conserved, the ICS activities of NtICS, NbICS, and OsICS were much lower than that of AtICS1 both *in vitro* and *in planta*. The molecular mechanisms underlying differences in the ICS activities were investigated and discussed.

## Experimental

### Molecular cloning of the coding sequences of *AtICS1, NtICS, NbICS*, and *OsICS*

To amplify the coding sequences, primers corresponding to the 5′- and 3′-ends of the coding sequences were designed based on the sequences in the databases (*AtICS1*, AY056055, and *OsICS*, AK120689). Because the coding sequences of *NtICS* and *NbICS* in the databases were partial, their 5′- and 3′-ends were identified by BLAST searches using the NCBI database (http://www.ncbi.nlm.nih.gov/BLAST/) and the Sol Genomics Network database (http://solgenomics.net/), respectively, based on sequence similarity to tomato (*Solanum lycopersicum*) ICS (DQ984132) and pepper (*Capsicum annuum*) ICS (AY743431). Total RNA was prepared from healthy leaves of *Arabidopsis* (accession Col-0), tobacco (cv. Samsun NN), *N. benthamiana*, and rice (cv. Nipponbare). The coding sequences were amplified by reverse-transcription PCR from RNA templates, cloned into cloning vectors such as pBluescript II SK (+) (X52328), and sequenced. The coding sequence of cloned *AtICS1* was identical with that of AY056055. The coding sequences of cloned *NtICS* and *NbICS* were identical with those in the databases. For the coding sequence of *OsICS*, two variant forms were obtained. The nucleotide sequence of one variant was identical with that of AK120689, but, compared with the other plant ICSs, it contained a 4-nt deletion, which resulted in the generation of a premature stop codon. However, the other variant did not contain this deletion. Because the variant with the 4-nt deletion was very rare, we used the variant without the deletion as *OsICS*. The nucleotide sequences of *NtICS, NbICS*, and *OsICS* have been deposited in the GenBank/EMBL/DDBJ database (accession numbers: LC222287–9).

### Construction of chimeric ICSs and ICSs with mutations

The construction of chloroplast TP of tobacco ribulose bisphosphate carboxylase-oxygenase small subunit (TP^SS^)-EntC and TP^SS^-IPL^PmsB^, EntC and IPL^PmsB^ with the chloroplast transit peptide (TP) from tobacco ribulose bisphosphate carboxylase-oxygenase small subunit, respectively, was described previously [27]. The TP^SS^-IPL^PmsB^-ICSΔTP constructs used in Figure 4A were produced by introducing a BamHI site between TP^SS^-IPL^PmsB^ and ICS without the putative chloroplast TP. The TP^SS^-OsICSΔTP construct used in Figure 6B,C was made similarly by introducing BamHI site between TP^SS^ and OsICS without the putative chloroplast TP. Chimeric ICSs, AOO, AAO, OOA, and OAA, shown in Figure 5, and A136 and A102 shown in Figure 6A were produced using the restriction enzyme sites present in AtICS1 (PstI and HindIII) and OsICS (AscI, AvaI, and BglI). The other chimeric ICSs were created by overlap PCR with primer pairs as follows: A63, GCAACGCCCGCGTCTCCACTGTACCAAGTG and CACTTGGTACAGTGGAGACGCGGGCGTTGC; TP^AtICS1^-OsICS (A47), CGCGCCGCACCCATTCATAGACATCGAACA and TGTTCGATGTCTATGAATGGGTGCGGCGCG; TP^OsICS^-AtICS1, TCTCCATCACAACCATTCATCGACAGCGAG and CTCGCTGTCGATGAATGGTTGTGATGGAGA; TP^AtICS1^-NtICS, GTCGCCTTGGCAACCATTCATAGACATCGAACATG and CATGTTCGATGTCTATGAATGGTTGCCAAGGCGAC; TP^AtICS1^-NbICS, GCCTTGGCACCCATTCATAGACATCGAACATGACT and AGTCATGTTCGATGTCTATGAATGGGTGCCAAGGC; 134O, GACAGGTGTTGTACTCTTGCAAGCTTCCTC and GAGGAAGCTTGCAAGAGTACAACACCTGTC; 108O, AACGGCTGGGCTCGGATGCAGAGCAGCCAATATTT and ATATTGGCTGCTCTGCATCCGAGCCCAGCCGTTTG; 73O, ACTTTCAGCTCCTCCAAAAAATCCAATAGG and CCTATTGGATTTTTTGGAGGAGCTGAAAGT; 48O, AATTCCGGCACCAGCATAGATCAATGCCCCAAGAC and TGGGGCATTGATCTATGCTGGTGCCGGAATTGTTG; and 21O, TGATACTGCAGTAACTTGGTGAACTGAGATATCTT and ATCTCAGTTCACCAAGTTACTGCAGTATCAAGAAC. Point mutations were introduced using a QuikChange site-directed mutagenesis kit (Stratagene, http://www.stratagene.com).

### Expression of recombinant ICS proteins in *E. coli*

The coding sequences without the putative chloroplast TP (Supplementary Figure S1) were amplified by PCR with NdeI at the 5′-end and XhoI or SalI at the 3′-end, and cloned into the corresponding sites of a pET28a vector (Novagen), allowing for the production of each ICS with a His_6_ tag at the N-terminus. The resulting constructs were used to transform *E. coli* strain SHuffle T7 (New England Biolabs). Transformed cells were cultured at 30°C to mid-log phase, and the expression of recombinant proteins was induced by the addition of isopropyl β-d-thiogalactopyranoside at the concentrations indicated: 0.4 mM for AtICS1, OsICS, and their derivatives; 0.04 mM for NtICS; 0.01 mM for NbICS; and 0.5 mM for vector control and EntC. The cells were collected using a brief centrifugation after an 18-h culture at 20°C, resuspended in extraction buffer A (0.1 M Tris/HCl, pH 7.5, 1 mM DTT, 10% glycerol) containing cOmplete, EDTA-free protease inhibitor cocktail (Roche) and sonicated on ice. After centrifugation, the supernatants were desalted using a PD MiniTrap G-25 desalting column (GE Healthcare) equilibrated with buffer A in accordance with the manufacturer’s recommendations, and used as a crude protein fraction.

### Plant materials and plant growth conditions

*N. benthamiana* was grown in a temperature-controlled growth room maintained at 25°C with a 16-h light/8-h dark cycle. Plants at approximately 5 weeks old were used for the experiments.

### Expression of recombinant ICS proteins in *N. benthamiana* leaves

The entire coding sequences with or without three FLAG tags (DYKDHDGDYKDHDIDYKDDDDK) at the C-terminus were cloned into the multiple cloning sites of the binary vector pEl2Ω [28]. The constructs were used to transform *Agrobacterium tumefaciens* strain GV3101 by electroporation. Transformed cells were cultured at 30°C to mid-log phase and collected by centrifugation. After washing with 10 mM MES-NaOH (pH 5.6), 10 mM MgCl_2_, and 150 μM acetosyringone, the cells were suspended with the same solution to A_600_ = 0.1. After a 2-h incubation at room temperature, the cells were infiltrated into the leaves of *N. benthamiana* using a syringe without a needle.

For the ICS activity assays *in vitro*, leaves infiltrated with *Agrobacterium* were collected at 2 days after infiltration and ground in buffer A containing cOmplete, EDTA-free protease inhibitor cocktail (Roche). After centrifugation, the supernatants were desalted using a PD MiniTrap G-25 desalting column (GE Healthcare) equilibrated with buffer A in accordance with the manufacturer’s recommendations, and used as a crude protein fraction.

For ICS activity assays *in planta, Agrobacterium* cells carrying each ICS gene were mixed with different volumes of *Agrobacterium* cells carrying the TP^SS^-IPL^PmsB^, as indicated in Figure 4B. Two days later, the levels of total SA were determined as described previously [29].

### ICS activity assay *in vitro*

ICS activity *in vitro* was assayed as described previously [30]. Briefly, the crude protein fraction was incubated at 30°C for 1 h in buffer A containing 10 mM MgCl_2_ and 1 mM barium chorismate (Sigma C-1259). The isochorismate produced was converted into SA, and the amount of SA was measured as described previously [29]. The linearity of the reaction was confirmed using various volumes of crude protein fractions in all assays except for EntC expressed in *E. coli*.

### Immunoblotting analyses

Proteins were separated by SDS/PAGE and then transferred on to PVDF membranes (Millipore Corp.). After blocking with nonfat milk, the membranes were probed with 0.04 μg. ml^−1^ anti-His_6_ antibody (Life Technologies) or 5000^−1^ anti-DYKDDDDK antibody (Wako). After washing, the membranes were incubated with an alkaline phosphatase-labeled secondary antibody. The antigen–antibody complexes were detected by hydrolysis of nitro blue tetrazolium/5-bromo-4-chloro-3-indolyl phosphate as the substrate.

### Tertiary structure prediction of AtICS1 by homology modeling

A structural model of the chorismate-binding domain (279–558) of AtICS1 was predicted by homology modeling using the SWISS-MODEL program (http://swissmodel.expasy.org/) [31–33]. The structure of menaquinone-specific ICS *E. coli* MenF (PDB ID: 2EUA) [22] was used as a template (amino-acid sequence identity, 34%). Isochorismate and Mg^2+^ were appropriately located in the model by reference to the structure of the enterobactin-specific ICS EntC (PDB ID: 3HWO) [23]. Structural figures were prepared using PyMOL (DeLano Scientific).

## Results

### Comparison of deduced amino acid sequences of plant *ICS* genes

Because the coding sequences of *NtICS* and *NbICS* in the databases were incomplete, their full-length coding sequences were cloned based on similarity with those of tomato ICS (*SlICS*, DQ984132) and pepper ICS (*CaICS*, AY743431). Compared with other plant ICSs, the coding sequence of *OsICS* in the database (AK120689) contained a 4-nt deletion, which resulted in the generation of a premature stop codon. When we cloned the coding sequence of *OsICS*, two variant forms of *OsICS* were obtained. The nucleotide sequence of one variant contained the same 4-nt deletion as the database sequence, whereas that of another variant did not. Because the number of copies of the variant with the 4-nt deletion was very low, we used the variant without the deletion as *OsICS*. The deduced amino acid sequences of NtICS, NbICS, and OsICS were compared with those of plant ICSs. Multiple sequence alignment analysis revealed that the overall sequences were well conserved, and all ICSs had a putative chloroplast TP and a chorismate-binding domain at the N-terminal end and in the C-terminal half, respectively (Supplementary Figure S1). Amongst eight amino acid residues essential or important for ICS activity in EntC and/or MenF [22,23], six were completely conserved in all the plant ICSs analyzed here except Lys^364^ and Met^379^ of PtICS, which were glutamic acid and leucine, respectively, in the other ICSs. The remaining two amino acid residues were phenylalanine in EntC, but were tyrosine (496th and 528th amino acids of AtICS1) in all the plant ICSs shown here. Similarly, amongst six amino acid residues required for anthranilate synthase activity in *Salmonella typhimurium* TrpE [34], five were completely conserved in all the plant ICSs shown here, except for Glu^412^ of OsICS, which was aspartic acid in TrpE. The remaining amino acid, threonine in TrpE, was alanine (472nd amino acid of AtICS1) in all the analyzed ICSs. The conservation of the overall structures and key amino acid residues suggested that *NtICS, NbICS*, and *OsICS* encode ICS proteins similar to *AtICS1*.

### Comparison of the ICS activity of the recombinant proteins expressed in *E. coli*

**Figure 2:**
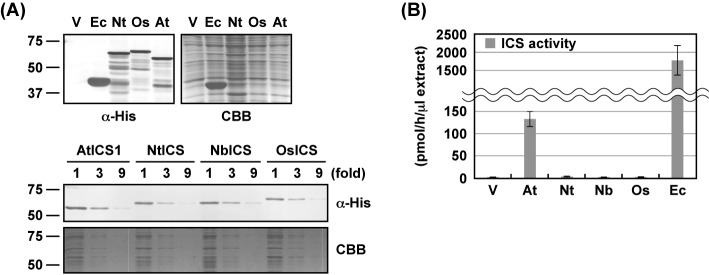
Comparison of ICSs expressed in *E. coli* (**A**) Recombinant proteins of EntC (Ec), NtICS (Nt), OsICS (Os), AtICS1 (At), and NbICS (Nb) with His_6_ tags at the N-terminus were expressed in *E. coli*. The crude protein fractions were prepared from the cells, and the production of recombinant proteins was confirmed by immunoblotting analyses using an anti-His_6_ tag antibody (α-His). *E. coli* transformed with an empty vector were used as a control (V). As a loading control, parallel gels were stained with Coomassie Brilliant Blue (CBB). To verify that similar levels of the recombinant proteins were expressed, serially diluted (fold) crude protein fractions were analyzed. (**B**) ICS activity of the crude protein fractions was measured. Values are means with S.D. of three independently prepared crude protein fractions.

To compare the ICS activity of NtICS, NbICS, and OsICS with that of AtICS1, their recombinant proteins without putative TPs were expressed in *E. coli* as N-terminal His_6_-tagged proteins. As negative and positive controls, an empty vector and a vector carrying EntC were also introduced into *E. coli*. Crude protein fractions were obtained from the transformed cells, and expression of the recombinant proteins was confirmed by immunoblotting analysis with an anti-His_6_ tag antibody. Except for EntC, similar levels of the four plant ICS proteins were expressed (Figure 2A). ICS activity was measured using the crude protein fractions. As expected, AtICS1 and EntC showed activity that was much higher than the empty vector control (Figure 2B). In contrast, the crude protein fractions containing NtICS, NbICS, or OsICS showed no significant difference from the empty vector control.

### Comparison of ICS activity of the recombinant proteins expressed in *N. benthamiana* leaves

**Figure 3:**
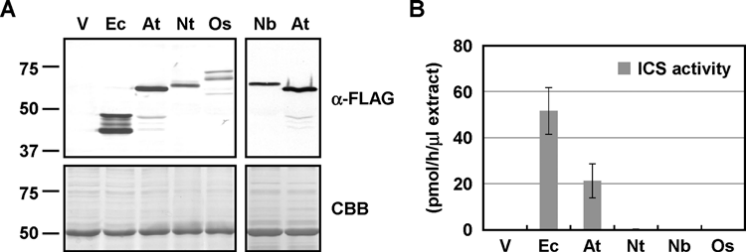
Comparison of ICSs expressed in *N. benthamiana* leaves (**A**) *Agrobacterium* cells (A_600_ = 0.1) carrying each ICS with three FLAG tags at the C-terminus were infiltrated into the leaves of *N. benthamiana. Agrobacterium* carrying an empty vector was used as a control (V). Two days later, the accumulation of ICS proteins was detected by immunoblotting analyses using an anti-FLAG tag antibody (α-FLAG). As a loading control, parallel gels were stained with Coomassie Brilliant Blue (CBB). (**B**) ICS activity of the crude protein fractions was measured. Values are means with S.D. (*n*=3–11). Abbreviations: At, AtICS1; Ec, TP^SS^-EntC; Nb, NbICS; Nt, NtICS; Os, OsICS.

When expressed in *E. coli*, NtICS, NbICS, and OsICS showed little ICS activity (Figure 2B). This result suggested that they might require post-translational modification for activation or they were not folded properly in *E. coli*. Therefore, recombinant proteins of plant ICSs and EntC with three FLAG tags at their C-terminus were expressed in *N. benthamiana* leaves by agroinfiltration. In plants, ICS functions in the chloroplast, so TP^SS^ was introduced into the N-terminus of EntC. Immunoblotting analyses with an anti-FLAG antibody confirmed the production of all the proteins, but their levels and band patterns differed (Figure 3A). EntC was detected as two major and several minor bands. The migration of two major bands (46.8 and 42.2 kDa) corresponded well with the predicted molecular mass of EntC with (47.3 kDa) and without the TP (41.6 kDa). Because EntC was fused to a heterologous chloroplast TP, it may not have been cleaved efficiently. Plant ICSs, except for OsICS, were detected as a single major band with a good correlation with the predicted molecular masses of the mature proteins. OsICS was detected as three bands with distinct intensities. From the migration of the middle (66.2 kDa) and lower bands (57.0 kDa) on the gel, they seemed to be pre-protein and mature forms of the protein (predicted to be 63.1 and 58.3 kDa, respectively). However, in subsequent experiments, it was suggested that the middle band corresponded to the mature protein, and the upper band was likely to be the pre-protein (Figure 6). The faint, lower band could be a degradation product of OsICS. The levels of EntC and AtICS1 were higher than those of NtICS, NbICS, and OsICS (Figure 3A). ICS activity was measured using the crude protein fractions. Again, AtICS1 and EntC showed ICS activity, but NtICS, NbICS, and OsICS showed little activity, which was not significantly different from the empty vector control (Figure 3B).

### Comparison of the ICS activity of the recombinant proteins *in planta*

**Figure 4:**
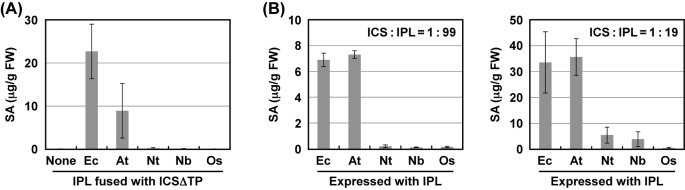
Comparison of ICSs *in planta* (**A**) *Agrobacterium* cells (A_600_ = 0.1) carrying each artificial SAS construct (TP^SS^-IPL^PmsB^-ICSΔTP) were infiltrated into the leaves of *N. benthamiana. Agrobacterium* carrying TP^SS^-IPL^PmsB^ only was used as a control (none). Two days later, the levels of SA were determined. Values are means with S.D. (*n*=3). (**B**) *Agrobacterium* cells (A_600_ = 0.1) carrying each ICS were mixed with 99 volumes (left) or 19 volumes (right) of *Agrobacterium* cells (A_600_ = 0.1) carrying TP^SS^-IPL^PmsB^ (IPL). Mixtures were infiltrated into the leaves of *N. benthamiana*. Two days later, the levels of total SA were determined. Values are means with S.D. (*n*=3). Abbreviations: At, AtICS1; Ec, EntC and TP^SS^-EntC; Nb, NbICS; Nt, NtICS; Os, OsICS.

*In vitro* ICS assays failed to detect activity with NtICS, NbICS, and OsICS expressed in *E. coli* and *N. benthamiana* leaves (Figures 2B and 3B). Because isochorismate is a precursor of phylloquinone, which is required for the function of the photosystem I complex, all green plants are thought to have ICS activity. These results suggested that NtICS, NbICS, and OsICS have activity in plant cells, but they are rapidly inactivated after extraction from the cells. Therefore, we tried to measure their ICS activity *in planta*. Because isochorismate is unstable and converted into other metabolites such as phylloquinone, it is difficult to measure *in planta* ICS activity by quantitating the levels of isochorismate in plant cells. In contrast with isochorismate, SA is stored in the form of SA-glucoside in plant cells for a long time. It has been reported that an artificial SAS can be created by fusing ICS with IPL, and overexpression of the artificial SAS resulted in the accumulation of SA [27,35]. We applied this artificial SAS system to measure the *in planta* ICS activity. IPL encoded by *PmsB* of *Pseudomonas fluorescens* [27] was fused to the N-terminus of each ICS without a putative chloroplast TP, resulting in IPL^PmsB^-ICSΔTP. Because chorismate is produced in chloroplasts, TP^SS^ was introduced at the N-terminus of fusion proteins, resulting in TP^SS^-IPL^PmsB^-ICSΔTP. The artificial SASs (TP^SS^-IPL^PmsB^-ICSΔTP) were expressed in *N. benthamiana* leaves by agroinfiltration, and the levels of SA produced were measured 2 days later. As shown in Figure 4A, the expression of artificial SASs containing EntC or AtICS1 resulted in higher levels of SA accumulation. In contrast, the expression of artificial SASs containing NtICS, NbICS, or OsICS led to very low levels of SA accumulation, which were not significantly different from those with TP^SS^-IPL^PmsB^ alone. This result suggested that NtICS, NbICS, and OsICS showed little ICS activity even in plant cells; however, we cannot rule out the possibility that these proteins were inactivated by the fusion with TP^SS^-IPL^PmsB^. As a next attempt, ICSs were expressed with TP^SS^-IPL^PmsB^. This method has several disadvantages. First, isochorismate produced by ICS can be used not only by IPL but also by other enzymes. Second, if ICS activity is higher than IPL activity, only part of the isochorismate will be converted into SA. These factors may have led to an underestimation of ICS activity. Third, as shown later, TPs affected the levels of protein accumulation *in planta*. To overcome these difficulties, *Agrobacterium* cells carrying ICS were mixed with excess amounts of those carrying TP^SS^-IPL^PmsB^, as shown in Figure 4B. When ICS and IPL were expressed at a ratio of 1 to 99, EntC and AtICS1 showed ICS activity, whereas the others showed little activity, which is not significantly different from that of TP^SS^-IPL^PmsB^ alone. Unlike the results obtained using the artificial SAS assay (Figure 4A), EntC and AtICS1 showed similar levels of activity. This difference suggested that the conversion of isochorismate into SA by IPL was rate-limiting even in these conditions, and ICS activity of EntC, and likely that of AtICS1, is underestimated. To increase the sensitivity of the assay, the ratio of ICS to IPL was changed to 1 to 19. In these conditions, the expression of NtICS or NbICS with IPL resulted in significant increases in the levels of SA, indicating that they had ICS activity. In contrast, the expression of OsICS with IPL did not increase the levels of SA, even when proportion of *Agrobacterium* cells carrying OsICS was increased up to 50% (Figure 6C).

### Chimeric analysis of AtICS1 and OsICS

**Figure 5:**
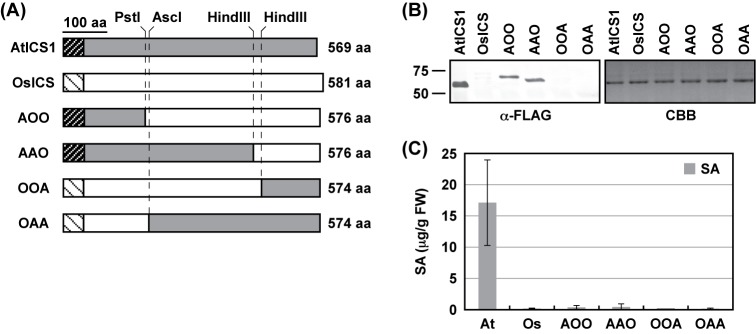
Chimeric analysis of AtICS1 and OsICS (**A**) Schematic representation of AtICS1 and OsICS chimeras. Putative chloroplast TPs are shown as hatched boxes. Restriction enzyme sites used to create chimeric proteins are shown at the top. (**B**) *Agrobacterium* cells (A_600_ = 0.1) carrying each ICS with three FLAG tags at the C-terminus were infiltrated into the leaves of *N. benthamiana*. Two days later, the accumulation of ICS proteins was detected by immunoblotting analyses using an anti-FLAG tag antibody (α-FLAG). As a loading control, parallel gels were stained with Coomassie Brilliant Blue (CBB). (**C**) *Agrobacterium* cells (A_600_ = 0.1) carrying each ICS with three times FLAG tag at the C-terminus were mixed with *Agrobacterium* cells (A_600_ = 0.1) carrying TP^SS^-IPL^PmsB^. Mixtures were infiltrated into the leaves of *N. benthamiana*. Two days later, the levels of SA were determined. Values are means with S.D. (*n*=3). Abbreviations: At, AtICS1; Os, OsICS.

The results indicated that plant ICSs are quite different from each other in terms of activity (Figures 2B, 3B, and 4) and protein accumulation in *N. benthamiana* leaves (Figure 3A), despite their primary structures being similar to each other and their key amino acid residues are nearly completely conserved (Supplementary Figure S1). To identify regions responsible for the differences between AtICS1 and OsICS, four chimeric proteins were created between AtICS1 and OsICS (Figure 5A). They were expressed in *N. benthamiana* leaves by agroinfiltration with three FLAG tags at their C-terminus, and their protein levels were investigated by immunoblotting analysis with an anti-FLAG antibody. As shown in Figure 5B, chimeric proteins containing the N-terminal region of AtICS1 (AOO and AAO) accumulated to much higher levels than those containing the N-terminal region of OsICS (OOA and OAA). Next, ICS activity of chimeric proteins was measured *in planta*. They were expressed in *N. benthamiana* leaves with TP^SS^-IPL^PmsB^ by agroinfiltration, and the resulting SA levels were determined. Although proteins AOO and AAO accumulated to a high level, none of the chimeric proteins showed significant ICS activity (Figure 5C). There results indicated that the N-terminal and C-terminal regions of AtICS1 were responsible for the high levels of protein accumulation and ICS activity, respectively.

### Chloroplast TPs affect the levels of protein accumulation in *N. benthamiana* leaves

**Figure 6:**
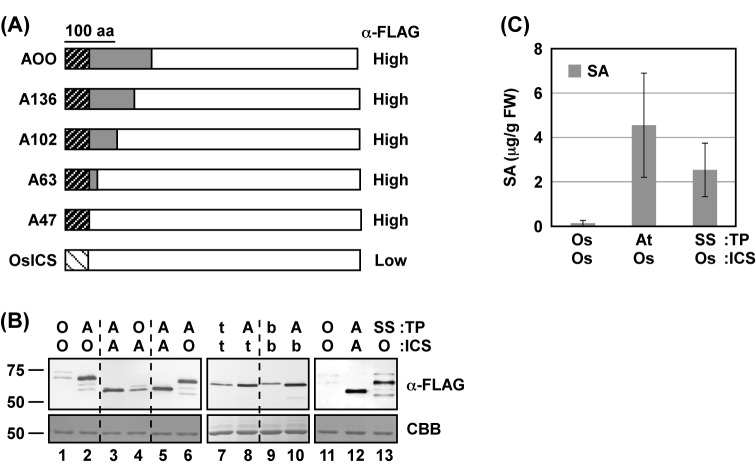
Putative chloroplast TPs affect the levels of protein accumulation in *N. benthamiana* leaves (**A**) Schematic representation of AtICS1 (gray) and OsICS (white) chimeras. Putative chloroplast TPs are shown as hatched boxes. *Agrobacterium* cells (A_600_ = 0.1) carrying each ICS with three FLAG tags at the C-terminus were infiltrated into the leaves of *N. benthamiana*. Two days later, the accumulation of ICS proteins was detected by immunoblotting analyses using an anti-FLAG tag antibody. The protein levels of the chimeric ICSs are summarized on the right (α-FLAG). (**B**) *Agrobacterium* cells (A_600_ = 0.1) carrying each ICS with three FLAG tags at the C-terminus were infiltrated into the leaves of *N. benthamiana*. Two days later, the accumulation of the ICS proteins was detected by immunoblotting analyses using an anti-FLAG tag antibody (α-FLAG). As a loading control, parallel gels were stained with Coomassie Brilliant Blue (CBB). (**C**) *Agrobacterium* cells (A_600_ = 0.1) carrying each ICS were mixed with *Agrobacterium* cells (A_600_ = 0.1) carrying TP^SS^-IPL^PmsB^. Mixtures were infiltrated into the leaves of *N. benthamiana*. Two days later, the levels of SA were determined. Values are means with S.D. (*n*=3). Abbreviations: A and At, AtICS1; b, NbICS; O and Os, OsICS; SS, tobacco ribulose-1,5-bisphosphate carboxylase/oxygenase small subunit; t, NtICS.

The region responsible for the difference in the protein levels of AtICS1 and OsICS in *N. benthamiana* leaves was mapped by shortening the AtICS1 sequence region in the chimeric proteins (Figure 6A). Immunoblotting analyses revealed that the chloroplast TP of AtICS1 (hereafter, TP^AtICS1^) was sufficient for the high levels of protein accumulation in *N. benthamiana* leaves (Figure 6A,B, lanes 1, 2, 5, 6). Conversely, replacement of TP^AtICS1^ with the TP of OsICS significantly reduced the accumulation of AtICS1 protein (Figure 6B, lanes 3, 4). The effect of TP^AtICS1^ was not specific to OsICS; replacement of TP of NtICS and NbICS with the TP^AtICS1^ resulted in increased accumulation of the proteins (Figure 6B, lanes 7–10). Similar effects have been reported for the TP of several proteins, and the best known is TP of ribulose bisphosphate carboxylase-oxygenase small subunit proteins [36]. Therefore, the effect of TP^SS^ on the protein levels of OsICS was investigated. As expected, replacement of the TP of OsICS with TP^SS^ increased the levels of protein accumulation (Figure 6B, lanes 11–13). Reflecting the increase in protein levels, OsICS with TP^AtICS1^ or TP^SS^ showed low but significant levels of ICS activity *in planta* (Figure 6C), indicating that *OsICS* encoded a functional ICS.

### Identification of amino acid residues required for the activity of AtICS1

**Figure 7:**
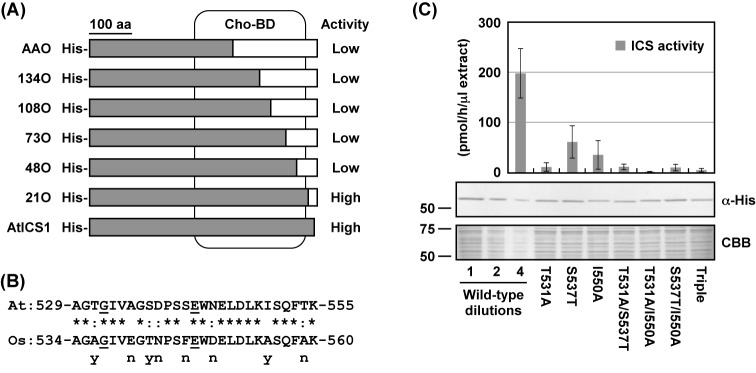
Identification of amino acid residues required for ICS activity of AtICS1 (**A**) Schematic representation of AtICS1 (gray) and OsICS (white) chimeras. Recombinant proteins of chimeric ICSs with a His_6_ tag (His) at the N-terminus were expressed in *E. coli*. The crude protein fractions were prepared from the cells, and their ICS activities was measured. The activities of the chimeric ICSs are summarized on the right (activity). (**B**) Alignment of the 529–555th amino acid sequence of AtICS1 with the corresponding region of OsICS. The asterisks (*) and colons (:) indicate identical and similar amino acid residues between AtICS1 and OsICS, respectively. Amino acid residues known to be essential for the anthranilate synthase activity of *S. typhimurium* TrpE are underlined. Each different amino acid of AtICS1 was replaced with the corresponding amino acid of OsICS, and the recombinant proteins of the AtICS1 mutants were expressed as an N-terminal His_6_-tagged protein in *E. coli*. Crude protein fractions were prepared from the cells, and their ICS activities were measured. Effects of single amino acid substitutions on the ICS activity of AtICS1 are summarized below the sequence of OsICS. n, no significant effect; y, significant effect. (**C**) Recombinant proteins of AtICS1 mutants with a His_6_ tag at the N-terminus were expressed in *E. coli*. The crude protein fractions were prepared from the cells, and their ICS activities were measured. Values are means with S.D. of three independently prepared crude protein fractions. The production of recombinant proteins was confirmed by immunoblotting analyses using an anti-His_6_ tag antibody (α-His). As a loading control, parallel gels were stained with Coomassie Brilliant Blue (CBB). Abbreviations: At, AtICS1; Cho-BD, chorismate-binding domain; Os, OsICS; Triple, T531A/S537T/I550A.

The region responsible for the difference in the levels of the ICS activity of AtICS1 and OsICS was mapped by shortening the OsICS sequence region in the chimeric proteins (Figure 7A). When expressed in *E. coli*, the recombinant protein 21O showed ICS activity similar to that of AtICS1, whereas the activity of 48O was below the detection limit (Figure 7A). The amino acid sequences of the corresponding differential region between 21O and 48O are highly conserved; only 8 out of 27 residues were different between AtICS1 and OsICS (Figure 7B). Each amino acid amongst the eight residues specific to AtICS1 was replaced with the corresponding one from OsICS, and the ICS activities of the recombinant proteins were examined. As summarized in Figure 7B (marked as ‘y’), replacement of Thr^531^, Ser^537^, or Ile^550^ of AtICS1 reduced its activity. The T531A mutant showed the lowest activity compared with the S537T and I550A mutants (Figure 7C). Moreover, the introduction of double and triple mutations compromised the ICS activity of AtICS1 almost completely. To investigate whether the three amino acid residues identified were sufficient for the high activity as well as AtICS1, the three amino acid residues of OsICS (Ala^536^, Thr^542^, and Ala^555^) were replaced with the corresponding residues of AtICS1. The recombinant protein of OsICS with the triple mutation was produced in *E. coli*, and its ICS activity was analyzed. However, the triple mutant of OsICS still showed little activity*. These results suggested that not only the three residues located in the C-terminal region, but also other amino acid residues in other regions are involved in the difference between the activities of AtICS1 and OsICS.

## Discussion

### Biochemical properties of plant ICSs and their role in SA biosynthesis

ICS catalyzes the formation of isochorismate, a precursor of primary and secondary metabolites such as phylloquinone and SA. In the present study, we showed that AtICS1 has much higher ICS activity than NtICS, NbICS, and OsICS (Figures 2–4), and its chloroplast TP has the ability to increase the accumulation of attached proteins (Figure 6). These biochemical properties of AtICS1 and the fact that the expression of *AtICS1* was strongly induced when the biosynthesis of SA is activated [11,12] matched well with its role in SA production in response to environmental stress. To cope with stress, especially to fight pathogens, it is important to induce stress responses by producing SA rapidly. In contrast, NtICS is unlikely to be involved in stress-induced SA production, because it showed much lower ICS activity than that of AtICS1 (Figures 2–4), and its expression was not induced after the activation of SA synthesis by stress treatment [18,19]. Rice constitutively contains much higher levels of SA than *Arabidopsis* [21], but the ICS activity of OsICS was much lower than AtICS1 (Figures 2–4). Considering that rice contains only a single *ICS* gene, SA could be produced through an ICS-independent pathway in rice. Consistent with our hypothesis, a recent report showed that 3-hydroxyacyl-CoA dehydrogenase, an enzyme involved in β-oxidation, is involved in SA production in rice roots [37]. The result that the ICS activity of NbICS was similar to that of NtICS and OsICS (Figures 2–4) was inconsistent with its role. Similar to AtICS1, NbICS is required for SA synthesis induced by biotic and abiotic stresses [17], and its expression is increased by INF1, an elicitor protein secreted by *Phytophthora infestans* [16]. On the other hand, it was consistent with the fact that the primary amino acid sequence of NbICS is quite similar to that of NtICS, with approximately 95% identity (Supplementary Figure S1). This inconsistency prompted us to analyze the expression pattern of *NbICS*, and we found that, in our reverse-transcription quantitative PCR analysis, *NbICS* was not induced by INF1^*^. Although the reasons for this discrepancy are unclear, the lack of a mock treatment in Shibata et al. 2010 [16] might be one reason. Moreover, a recent report showed that the expression of two ICS genes is not induced, but rather suppressed after the activation of SA synthesis in soybean, but their suppression by virus-induced gene silencing compromises SA accumulation induced by pathogen inoculation [38]. Because isochorismate is a precursor of phylloquinone, a component of the photosystem I complex, severe suppression of ICS caused several developmental defects, as observed in the double mutant of AtICS1 and AtICS2 [15,39] and in an *NbICS*-silenced plants [16]. These lines of evidence suggested that NbICS, unlike AtICS1, is unlikely to play a key role in SA biosynthesis, although it is involved indirectly.

### Structural mechanisms underlying the distinct biochemical properties of AtICS1 and OsICS

**Figure 8:**
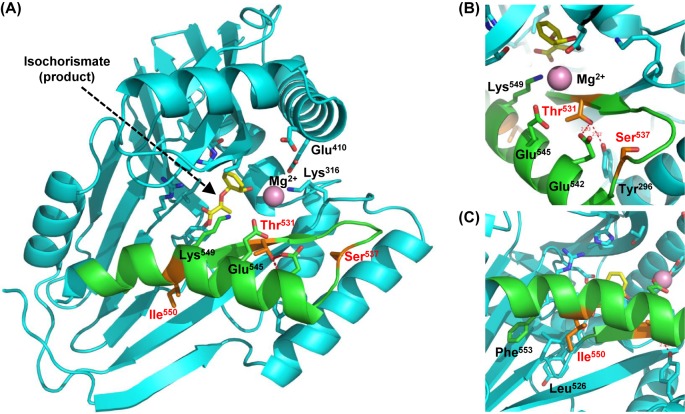
Tertiary structure model of AtICS1 predicted by homology modeling (**A**) Ribbon representation of the structure model of the chorismate-binding domain (279-558) of AtICS1. The region (529–555) responsible for the high activity of AtICS1 and the other regions are indicated in green and cyan, respectively. Important residues for enzymatic activity and structure are shown as stick models. Isochorismate is shown as a yellow stick model and the magnesium ion is a purple sphere. Critical mutation sites (Thr^531^, Ser^537^, and Ile^550^) for the activity are shown as orange stick models. (**B**) Close-up view of the AtICS1 model at the Thr^531^ and Ser^537^ sites. (**C**) Close-up view of the AtICS1 model at the Ile^550^ site.

Chimeric analysis between AtICS1 and OsICS revealed several structural mechanisms underlying their distinct biochemical properties. First, putative chloroplast TPs were shown to determine the levels of protein accumulation in *N. benthamiana* leaves (Figure 6). TP^AtICS1^ increased the protein levels of several ICSs, and its effect on the levels of OsICS accumulation was similar to that of TP^SS^ (Figure 6B,C). The TP of ribulose bisphosphate carboxylase-oxygenase small subunit proteins has been used to increase the levels of foreign protein accumulation in transgenic plants [36,40]. Although the effect of TP^AtICS1^ on proteins other than ICS has not been tested, it might be an attractive alternative to the TP of ribulose bisphosphate carboxylase-oxygenase small subunit proteins. Second, three amino acid residues (Thr^531^, Ser^537^, and Ile^550^) located in the C-terminal region of AtICS1 were required for its ICS activity (Figure 7C). To predict the roles of these amino acid residues, a tertiary structural model of the chorismate-binding domain (279-558) of AtICS1 was constructed by homology modeling with the structures of *E. coli* ICSs (MenF and EntC) as templates. The structural model of AtICS1 suggest that Thr^531^, Ser^537^, and Ile^550^ are close to the active site with the product isochorismate and Mg^2+^ (Figure 8A). The Thr^531^ residue forms hydrogen bonds with Glu^542^ and Tyr^296^ (Figure 8B), suggesting that the T531A mutation caused the loss of the two hydrogen bonds and destabilized the structures around it. The Ser^537^ residue is located in a loop near the active site (Figure 8B), implying that the S537T mutation caused destabilization of the loop and decreased the activity. In addition, the Ile^550^ residue contributes to hydrophobic interactions with Leu^526^ and Phe^553^ (Figure 8C). The I550A mutation reduced the hydrophobic interactions and destabilized the structure in the α-helix around the active site. Therefore, these mutations have serious consequences for the ICS activity of AtICS1. All three amino acid residues are conserved in AtICS2 which shows near-identical enzymological properties with AtICS1 [26], whereas Ile^550^ is replaced with threonine in NtICS and NbICS (Supplementary Figure S1), suggesting that Ile^550^ is responsible for the differences in the activity of AtICS1, NtICS, and NbICS (Figures 2–4). Third, these three amino acid residues are involved in the activity of AtICS1, but they are not sufficient to explain the difference in activity between AtICS1 and OsICS. Little ICS activity was detected with the OsICS mutant in which the three amino acid residues (Ala^536^, Thr^542^, and Ala^555^) were replaced with the corresponding ones from AtICS1 (Thr^531^, Ser^537^, and Ile^550^)^*^, suggesting that amino acid residues in other regions are also involved in the differences between the activities of AtICS1 and OsICS. Identification of such regions and residues should be a subject of subsequent analyses. To further understand the structural mechanisms underlying the differences between AtICS1 and OsICS, it will be required to determine their tertiary structures in a future analysis because no structural data for plant ICSs has yet been reported.

It has been suggested that the function of ICSs is regulated mainly at the transcriptional level in plants. For example, AtICS1 and AtICS2 show near-identical enzymological properties [26], but the transcript levels of *AtICS1*, but not *AtICS2*, are increased by stress conditions, and *AtICS1* is mainly required for the accumulation of SA induced by stress conditions [11,12]. Populus has a single *ICS* gene, which undergoes extensive alternative splicing, and the majority of the alternative transcripts encode nonfunctional proteins due to premature stop codons or multiexon skipping [41]. In this study, however, we revealed that plant ICSs show quite distinct biochemical properties despite their sequence similarities. These differences in transcriptional regulation and biochemical properties of plant ICSs may reflect their roles in primary (e.g. phylloquinone) and secondary (e.g. SA) metabolite production in their respective plant species.

## Supporting information

## Footnotes

* S. Katou (unpublished results)

## Abbreviations

DHBA: 2,3-dihydroxybenzoic acid
ICS: isochorismate synthase
IPL: isochorismate pyruvate lyase
SA: salicylic acid
SAS: salicylate synthase
TP: transit peptide
TP^SS^: chloroplast TP of tobacco ribulose bisphosphate carboxylase-oxygenase small subunit
