# Supplementary material for: Comparative analysis of plant isochorismate synthases reveals structural mechanisms underlying their distinct biochemical properties
Source: Biosci Rep. 2018 Mar 9;38(2):BSR20171457. doi: 10.1042/BSR20171457 (PMC5843753; doi:10.1042/BSR20171457)
Supplement: Supplementary file 1 [file bsr20171457_Supp1.pdf]

```
AtICS1 1   --MASLQFSSQFLGSNTKTHSS---IISISRSYSP-------TPFTRFSRKKYESCSMSMNGCDGDFK--TPLGTVETRTMTAVLSPAAA
AtICS2 1   --.....C.FH...T.P.KYNPSSIFQ.Y..TSFT-------KLSS.V..QRFLR.TL.....EA.H.--A.........LST.P.....
CaICS  1   --..VGARHCTLRFMEQLEST.LMKCSLS.SPLYQK---QYVHF.NS-.QRYSQC..L.....Q..PR--A.I..I....LP..S...L.
SlICS  1   --..VGVRHCTIRFMELES-..LMKCLLL.SPLYRK---QSVHFSNSSTQRYNQC..L.....Q..SR--A.I.I.....LP..S...L.
NtICS  1   --..VGAKHCTLR-----ES.-LMKCSLL.SPLYGK--QSIHFSNSSQR--YHQ...L.....Q..PR--A.I..I....LP..S...L.
NbICS  1   --..VGAKHCTLR-----ES..LVKWSLL.SPLYGIGKQSIHFSNSTQRSYHQSC..L.....Q..PR--A.I..I....FP..S...L.
CrICS  1   --...ITGHCVAHFTDLS.RK.SFFSN.NNN.SLFR---RKS.NIVTRKKYIFC.T.L.....N..PR--A.V..I....LP..ST..L.
PtICS  1   MAT.T.ARH.LAHFMDLESIKYSIAAQPV..R------QSLHLFYH.CYYH.PC.VVV.....Q.NPRGRV.I.SI....FP..T...L.
OsICS  1   --..PPPPPRTLSP.SSSPSLRSSGRRPLPSARLVG-----------RWVQRP...L.....GAAVAGAGAVAVR...ALP.AGAAGD.
              *                                                         :*****  .        .:.   ***::.:. :.. *

AtICS1 77  TERLISAVSELK-SQPPSFSSGVVRLQVPIDQQIGAIDWLQAQN--EIQPRCFFSRRSDVGRPDLLLDLANENGNG------NGNGTVSS
AtICS2 80  .....T...D..-....P....I.......E.K.......H...--..L..S.......S......Q.FSSD..--------------..
CaICS  83  M...N..I.D.VK.E..PY...TI..E...EE..E.LE..H...-HLLL......G.RPPTEIMCING------------------T..
SlICS  83  M...N..I.DMIK.D..PYD..II..E...EE..ESLE..H...-HLLL......G.R-.ASEMCING------------------A..
NtICS  77  M...N..I.D.LK.E..PYH..II..E...EE..E.LE..H...-HVLL......G.RAAVDSEMCFIDHTTK----------R...SH.
NbICS  82  M...N..I.D.VK.E..PYN..II..E...EE..E.LE..H...-HVLL......G.RPAVE.EMCFN-----------------..SH.
CrICS  84  M...S...AN..STL.-.AQ..II..E...EEH.E.L...HS.DQKNLL...Y..G..Q.TFS.FTSNDLTNRNGSAANGHLQRIS.S.D
PtICS  85  .DT.NL.I....-AN..L.T..IL......Q...E.....HS.H--Q.H......G.RQSKDFTEVTNG----------------NGYQK
OsICS  77  VGQ.RE..AA.EAAD..ASP..II.IE..VR.RGD.VE..H..G--DLRA.....A.AAAPL.ECPALAIASA...NGNG--A.GVGGEQ
              *  *::  :   : *     ** :*::**: ::  :::**::*     :  .*.:** *                        .

AtICS1 158 DRNLVSVAGIGSAVFFRDLDPFSHDDWRSIRRFLSSTSPLIRAYGGMRFDPNGKIAVEWEPFGAFYFSVPQVEFNEFGGSSMLAATIAWD
AtICS2 153 .H.P.........................K.........L...T........H..S...T.....D...........V...
CaICS  153 HSK......V......TH.R...F...A......KKC.......AI...ATAN..S..SA..S...M......D.LE...II.......
SlICS  152 HSK......V......TH.R...L...A......KKC.......AI...ATAN..S..SA..S...M......D.LE...II.......
NtICS  156 SSKV.....V......TH.R...F...A......KKC.......AI...ATAN.GP..SA..S...M......D.LE...VI...V...
NbICS  154 SSKV.....V......TH.R...F...A......KKC.......AI...ATAN.GP..NA..S...M......D.LE...VI...V...
CrICS  173 .K.......V....L..SPN...F...L..K....KNC.......AI...ARPH..P..KA..S...V......D.LH....I...V...
PtICS  156 SN.V.....V....L...VH..CYN..K..K....ANC.......AI...ARAN.SS......S...VI....LD.LD.C....T.....
OsICS  163 RQRP.....V.......GTE...LR...A.K...PRDC.......AI...ATSDHS....E..S...I.....Y..LEE..V..T.....
              . *****:****:*    **.  ** :*:***.  .*******.:***.  .  . **. **:*** :**** :*:   .*::*:*:***

AtICS1 248 DELSWTLENAIEALQETMLQVSSVVMKLRNRSLGVSVLSKNHVPTKGAYFPAVEKALEMINQKSSPLNKVVLARNSRIITDTDIDPIAWL
AtICS2 243 N.......................I.R..RE....I.V......SE...Y...NN...I.KD.H...S......S..............
CaICS  243 NAA...YQR..DE.RA.IW.L..IL.TVQKKIPHSHI.ART...G.SSWDH..KR..QI.SRND.V.I......ST.VV.AA....LT..
SlICS  242 NAA...YQR..D...A.IW.L...LITVQKKNPHSHI.ANT...G.ASWDH..NR..QI.SRNDPV.I......ST.VV.AA....LT..
NtICS  246 NAV.C.YQR...V..A.IW.....L.RVQKKIPHSHI.AST...G.ASWDQ..KR..QI.RRNDPM.I......ST.VV.AA....LT..
NbICS  244 NAV.C.YQRS.....A.IW.....L.RVQKKISRSHI.AST...G.ASWDQ..KR..QI.RRNNPM.I......ST.VV.AA....LT..
CrICS  263 NA..L.YQQ..V...T..E.....T.S...QDVSHT.LV..ANI.DRTSWDLTLNRV..E.GN.Y...T......R.QV..TS....L...
PtICS  246 NAF...W.Q.VD.VEA..T.I..N.L..SKEVTRSFI..NS...C.MYWDL...R..QI..RS....T......S.KFVIGN........
OsICS  253 .S....WQ..VKE..S.LQKI..SPI.VN.ST.QTTI.NL......ASWDL..T...Q..KG.QRE.V......C..Y....C...VEL.
           :  * * :.::  :. *: ::**      :  .         ::    ::* .  :  :: ..*: *   .    * ****** :: :      ***:  *

AtICS1 338 AQLQREGHDAYQFCLQPPGAPAFIGNTPERLFQRTQLGVCSEALAATRPRAASSARDMEIERDLLTSPKDDLEFSIVRENIREKLNGICD
AtICS2 333 .R..C..Q........................H.KH................GD.KV.E...................KT...
CaICS  333 .C.KV..EN.........HS..........Q..H.DR.SI......G..A.GG.ELL.VK..Q...S.A..HN..A....C..RR.EAV.S
SlICS  332 .C.KV..EN.........HS..........Q..H.DR.SI......G..A.GG.ELL.LN..Q...S.A..HN..A....C..RR.EAV.S
NtICS  336 .C.KV..EN.........QSA.........Q..H.DS.SI......G..A.GG.ELL.LK.GQ...S.A..HN..A....C..R..EAV.S
NbICS  334 SC.KV..EN.........QSA.........Q..H.DC.SI......G..A.GG.ELL.LK.GQ...S.G..HN..A....C..R..EAV.S
CrICS  353 SSFKAD.K.........HE...........Q..G.D..T.F.........A.GE.DSL.LQMAH..FS.....NH..A.......Q..DA..T
PtICS  336 .C..V..EN.........N.........AKQ..H.NC..IS...M.G..V.GG.M.L.LQ.QL...S....H...T...D...K..EAV..
OsICS  343 .C.KV..QN.....I...D....V..S...Q..H.KY.NIS.....G..A.GKTR.D.FQ.GQ...L.S.E.N..T...DS.KK..EM...
           : ::  :*.:*****:**   :.**:**:.::** *   *  : ***:*.** *.  :     :.::   **: * *:.  **:***: *:.:*.  :*

AtICS1 428 RVVVKPQKTVRKLARVQHLYSQLAGRLTKEDDEYKILAALHPTPAVCGLPAEEARLLIKEIESFDRGMYAGPIGFFGGEESEFAVGIRSA
AtICS2 423 ......H.S.........Q.KR....FN..T........C.V........Q................G....S.....
CaICS  423 S.LIE.K.AI..FS.....AR.R...QA....F...SSV.........Y.T.D..AF.S.T.M.........LV.W..............
SlICS  422 S.IIE.K.AI..FT.....AR.R...QA....F...SSV.........Y.T.D..AF.S.T.M..........V.W..............
NtICS  426 S.LIE.K.AI..FP.....AR.R...QT..E.F...SSI.........Y.T.D..AF.S.T.M..........V.W..............
NbICS  424 S.LIE.K.AI..FP.....AR.R...QT....F...SSI.........Y.T.D..AF.S.T.M..........V.W..............
CrICS  443 S.ETE.M.S....K.I......ARF...RS....F...SS..........F.M.D..KF.A.N.M....L.....V.....AQ.D.S.......
PtICS  426 .I..E.N.AI..FH.....AR...E.RS....F...SS..........F.T.......A.T.V..........V.W...G..........
OsICS  433 D...H.S.AL...P.....SA...A.MRN....FD..NT...S...........T....QF.QDY.I.......V.W...A...........
            :   .* *::**: *:*** :::  ..:   **:*:.** ::**:***** * *:** :* : * ****:*** :*:*** :*:*:******

AtICS1 518 LVEKGLGALIYAGTGIVAGSDPSSEWNELDLKISQFTKSIEYEATTSLQAIN-------------------------
AtICS2 513 ................S..N........E.........L.H.SALQPIN-----------------------------
CaICS  513 ................E...S.L..E..E..T.....LMKL..PLVTRGQRRIINQNQKVQRA--------------
SlICS  512 ................E...S.L..E..E..T.....LMKL..PLLTRGQSRIINQNQKGLSCLPHRI---------
NtICS  516 ................E...S.L..E..E..T.....LMKL..PLLTRREIRTINQNQKGLSCLQHHI---------
NbICS  514 ................E...S.L..E..E..T.....LMKL..PLLTRGEIRTINQNQKGLSCLQHHILVCLHHSF
CrICS  533 .IG.DA........L.V.E....AL..Q..E..A...M.LMKL..PALK----------------------------
PtICS  516 .A..............K..N..L..D..E..T.....LLKL.GPSRQKIE.SGIIN---------------------
OsICS  523 .LG..HST.V...A...E.TN..F..D.....A...A.LLQ.QEQHICLQEAENMGTVI------------------
           *   *.  .:*:*** *:* *::.: **:**:** *** *  :: :
```
